# Evaluating infant feeding practice guidelines for individuals with HIV: a pilot study using qualitative findings with healthcare workers and normalization process theory

**DOI:** 10.3389/frph.2026.1867104

**Published:** 2026-07-22

**Authors:** Julia D. López, Elaine Schmidt, Kneeshe Parkinson, Virginia McKay, Sydney M. Thayer, Amanda Zofkie

**Affiliations:** 1 Division of Infectious Diseases, Department of Medicine, Washington University School of Medicine, St. Louis, MO, United States; 2 Bursky School of Public Health, Washington University in St. Louis, St. Louis, MO, United States; 3 Brown School, Washington University in St. Louis, St. Louis, MO, United States; 4 Midwest D-CFAR, Washington University School of Medicine, St. Louis, MO, United States; 5 Department of Obstetrics & Gynecology, Division of Maternal-Fetal Medicine, Washington University School of Medicine, St. Louis, MO, United States

**Keywords:** breastfeeding/Chestfeeding, HIV, maternal and fetal medicine, normalization process theory, obstetrics and gynecology, pediatrics, pilot study, pregnant people

## Abstract

**Introduction:**

In 2023, the WHO and CDC updated HIV infant feeding guidelines, emphasizing the role of antiretroviral therapy (ART) in reducing breastfeeding/chestfeeding transmission risk among birthing people living with HIV (BPLWH). Despite this update, clinical integration remains inconsistent, shaped by provider knowledge, bias, patient engagement, and systemic barriers.

**Materials & methods:**

Between August 2025 and January 2026, we conducted 10 semi-structured, in-depth interviews with healthcare workers (HCWs) to assess their understanding of and engagement with updated HIV breastfeeding/chestfeeding guidelines. The Normalization Process Theory (NPT) informed the development of the interview guide and the thematic analysis. Transcripts were double-coded in Dedoose and thematic analysis was conducted deductively.

**Results:**

Healthcare workers generally accepted the guidelines as evidence-based, but their implementation varied across the four NPT constructs. While the guidelines were seen as logically coherent, OB-GYNs highlighted reproductive autonomy, whereas infectious disease and pediatric providers prioritized infant HIV susceptibility. Uncertainty about viral load thresholds led to variability among providers. Ethical motivation strongly supported implementation, but inconsistent training and low patient volumes reduced confidence. Institutional leaders and interdisciplinary workflows were identified as vital facilitators. Major obstacles included fragmented OB-GYN-to-pediatrics handoffs, services located in different areas, and structural patient burdens such as monitoring requirements and insurance issues, which the guidelines did not specifically address.

**Discussion:**

Systematically identifying how new breastfeeding/chestfeeding practices become embedded in healthcare among HCWs, and assessing where knowledge gaps, misunderstandings, and engagement barriers exist regarding the guidelines, will inform the development of a decision-making guide to enhance implementation of HIV breastfeeding/chestfeeding guidelines for pregnant people.

## Introduction

Since the start of the global HIV epidemic in the 1980s, nearly 10 million cases of perinatal HIV transmission have occurred globally (1), and about 3,000 people living with HIV (PLWH) give birth each year (2, 3). Before antiretroviral therapy (ART) was available, the rate of HIV transmission through breastfeeding/chestfeeding during the first two years of life was approximately 16% (4, 5). Preventive measures, including universal HIV screening and increased ART use during pregnancy, have effectively reduced perinatal HIV transmission. More recent research shows that the perinatal HIV transmission rate with breastfeeding/chestfeeding in the United States has fallen to less than 1%, with fewer than 150 cases reported annually since 2013 (3, 6, 7). This update also reflects a shift toward patient-centered counseling and shared decision-making in HIV care.

Prior to 2023, breastfeeding/chestfeeding was generally considered contraindicated for birthing people living with HIV (BPLWH) in the United States, with organizations such as the Department of Health and Human Services (DHHS), the American Academy of Pediatrics (AAP), and the Centers for Disease Control and Prevention (CDC) advising against this feeding method (8–10). Instead, formula feeding was strongly recommended as a safer alternative to breastfeeding/chestfeeding because it eliminated the risk of vertical transmission of HIV through breastfeeding/chestfeeding and was considered relatively accessible and affordable in a U.S. context (11, 12). In contrast, breastfeeding/chestfeeding has been recommended for BPLWH in many African regions due to limited access to clean water, the high cost of formula milk, and the fact that chestfeeding/breastfeeding improves morbidity and mortality rates, making it a more viable option in resource-constrained settings (13, 14). However, in January 2023 DHHS updated the U.S. national guidelines, titled *Recommendations for the Use of Antiretroviral Drugs During Pregnancy and Interventions to Reduce Perinatal HIV Transmission in the United States;* this update encouraged shared decision-making about infant feeding, including support for considering breastfeeding/chestfeeding for BPLWH who have sustained an undetectable viral load (15). The recommendations also highlighted how to manage cases of detectable viral load during breastfeeding/chestfeeding and offering additional avenues for support for BPLWH and their infants (15). This change aimed to recognize the benefits of breastfeeding/chestfeeding and the importance of individual choice, cultural considerations, and equity through the shared decision-making process between patients and healthcare workers (HCWs).

Despite this policy change, breastfeeding/chestfeeding guidelines are not always understood or consistently followed across clinical settings. Sociocultural, clinical, and logistical barriers contribute to disparities in care and leave both HCWs and patients uncertain about decision-making practices (16). For BPLWH, these challenges are compounded by stigma, bias, and unequal power dynamics among HCWs (17). Addressing these gaps in guideline implementation is therefore critical; therefore, to understand how breastfeeding/chestfeeding guidelines are adopted and sustained in practice, we must first identify the factors that shape their integration into clinical settings. This study aims to begin addressing these gaps in guideline implementation by understanding how HCWs interpret, engage with, and apply updated national guidelines; this knowledge can inform targeted strategies to overcome barriers and support consistent implementation.

We draw on Normalization Process Theory (NPT), a sociological framework that explains how new practices become embedded (or fail to become embedded) in healthcare (18). Using NPT, we analyze qualitative data to examine four constructs (coherence, cognitive participation, collective action, and reflexive monitoring) as they relate to HIV breastfeeding/chestfeeding guidelines among providers working with BPLWH. The findings presented here come from the formative phase of our project, Evaluating Infant Feeding Practice Guidelines for Individuals with HIV (ENGAGE-HIV).

## Materials & methods

### Study design and setting

In this qualitative study, recruitment, consent, and interviews were conducted with HCWs based in the Midwest (Missouri and Kansas). We completed semi-structured, in-depth interviews (IDIs) with 10 HCWs to assess their understanding of the HIV breastfeeding/chestfeeding guidelines (Supplementary File S1). Purposive sampling was used to maximize variation across provider types, clinical specialties, geographic regions, and institutional contexts, and the sample size was determined by qualitative analysis saturation. Eligibility criteria included: (1) being over the age of 18, (2) identifying as one of the following HCWs: OB/GYNs, Doulas, Midwives, Pediatric Infectious Disease Specialists, Adult Infectious Disease Specialists, Lactation Consultants, Community Health Workers, and Nurses working in settings where BPWLH receive care (i.e., infectious disease clinics, obstetric practices, postpartum units, and community health centers), and (3) having worked with BPLWH in the last 12 months. Participants were recruited by sending recruitment flyers through professional networks and to major local institutions servicing BPLWH. The study was approved by the Washington University in St. Louis Institutional Review Board (IRB #202503004). Participant confidentiality was maintained through the de-identification of all transcripts and the use of role-based identifiers in reporting the data.

### Theoretical framework

We grounded the IDIs in NPT, which provides the organizing framework for data collection and analysis (18). For this study, we structured our interview questions around NPT's four core constructs: *coherence (sense-making work)*, which captures the degree to which individuals understand and differentiate a new practice from existing work; cognitive participation *(engagement work)*, which captures the relational work involved in engaging with and enrolling others in a new practice; collective action *(operationalizing work),* which captures the operational work that individuals and groups do to implement and sustain a practice in everyday clinical contexts; and reflexive monitoring *(appraisal work),* which captures the formal and informal ways in which individuals and groups assess whether a practice is worthwhile and how it affects them and others (18). These constructs were used because they represent the foundational conditions for providers to adopt and implement breastfeeding/chestfeeding guidelines.

### Data collection

Semi-structured interviews were conducted with participants by a member of the research team (author ES) through HIPAA-encrypted Zoom. An interview guide was developed iteratively and organized around NPT constructs, probing providers' understanding of current breastfeeding/chestfeeding guidelines for BPLWH, how they distinguish these recommendations from general infant feeding guidance, and what facilitates or impedes their engagement with guideline implementation in practice. The lead investigator (author JDL) wrote the first interview guide, and after the first interview, which was proctored by ES, probing questions were added to elicit more in-depth responses. Probing questions were drafted by ES and edited and approved by JDL. All interviews were audio-recorded with participants' consent, transcribed verbatim using the transcription software Trint, and verified for accuracy.

Participants were asked to complete a demographic survey that included questions about primary area of practice, time in current role, primary work setting, age, race/ethnicity, and related work with BPLWH, including the question: “How many birthing parents living with HIV have you served who were pregnant or postpartum in the past 12 months?”

### Analysis

Transcripts were analyzed using a framework analysis approach, with NPT serving as the *a priori* analytic framework. A codebook was developed deductively based on the four NPT constructs and refined inductively through close reading of early transcripts. Two members of the research team (authors JDL and ES) independently coded a subset of transcripts (*n* = 3; 30% of all transcripts). Intercoder reliability was assessed using Cohen's kappa, which demonstrated substantial agreement (*κ* = .88). The remaining transcripts were double-coded with ongoing review by the research team (authors JDL and ES). The coded data were used to identify patterns, and illustrative codes helped highlight the patterns observed in the interviews. ES and JDL brought ongoing data collection and themes to co-authors in monthly team meetings for discussion.

## Results

**Figure 1:**
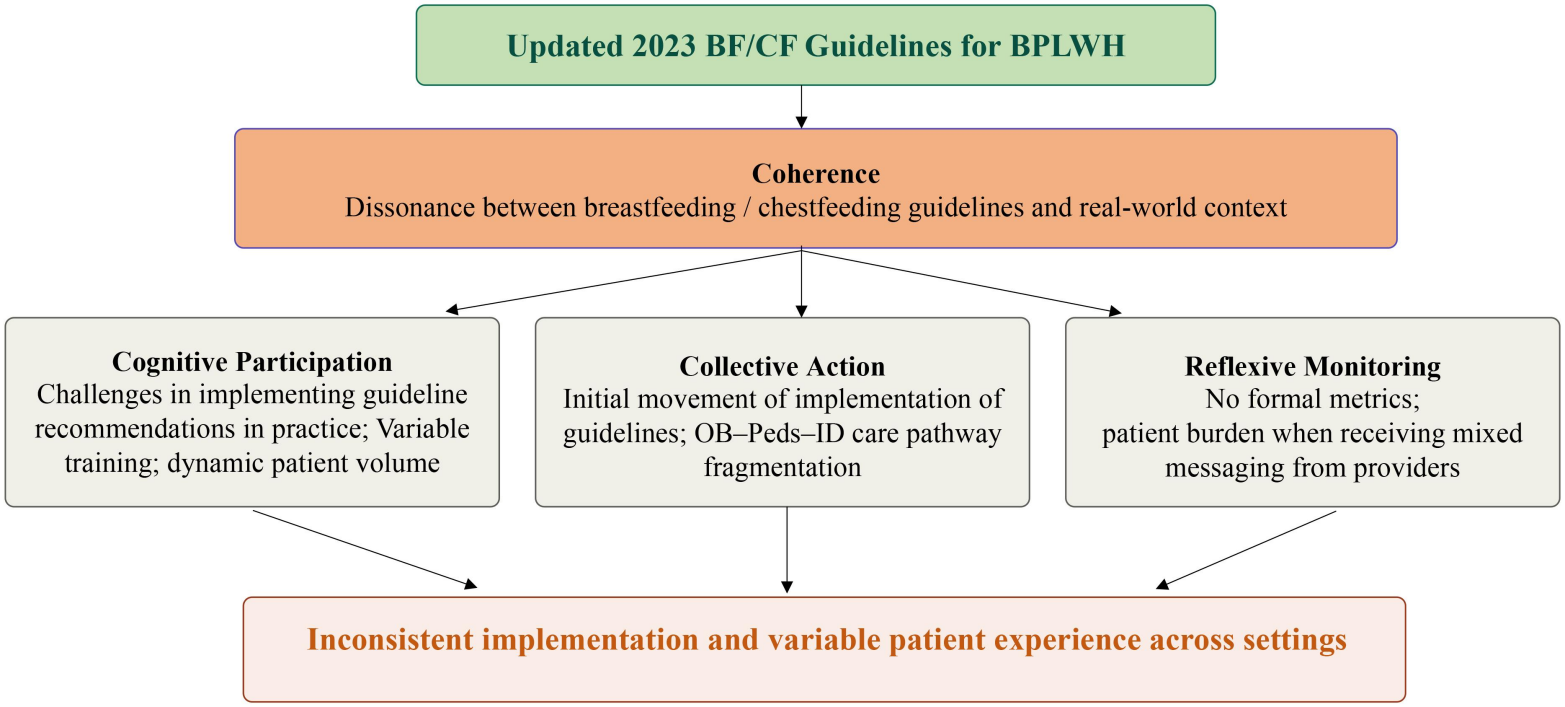
Applying NPT to breastfeeding/chestfeeding guideline implementation among HCWs caring for BPLWH.

The demographic information for study participants is outlined in Table 1, which includes 10 HCWs (7 Obstetricians, 1 Disease Intervention Specialist, 1 Pediatric Infectious Disease Clinical Nurse Coordinator, and 1 Pediatric Infectious Disease Physician). All participants were in the Midwest, and all but the Disease Intervention Specialist were from the same academic medical center. Each HCW completed a demographic survey and took part in an audio-recorded, one-hour Zoom IDI. The shortest interview lasted 33 min after informed consent, and the longest lasted 1 h and 10 min after informed consent. The average and median interview lengths were 49 min. The study is based on the NPT framework, and its findings are presented below with relevant quotations. The results are organized and presented under NPT concepts of (1) coherence, (2) cognitive participation, (3) collective action, and (4) reflexive monitoring (Figure 1). Additional quotations from the study can be found in Supplementary File S2.

**Table 1:** Participant characteristics (*n* = 10).

Current Role	Primary Area of Practice	Time in Current Role (years)	Primary Work Setting	Age range (years)	Race/Ethnicity	BPLWH served in the past 12 months^a^
Disease Intervention Specialist	HIV Care	1 to 3	Public Health Department	25–34	Black/African American	6 to 10
Physician (MD/DO)	Obstetrics and Gynecology	4 to 9	Academic medical center	35–44	Asian	1 to 5
Physician (MD/DO)	Obstetrics and Gynecology	10 to 19	Academic medical center	35–44	White/Caucasian	6 to 10
Physician (MD/DO)	Obstetrics and Gynecology	<1 year	Academic medical center	25–34	Asian	6 to 10
Physician (MD/DO)	Obstetrics and Gynecology	1 to 3	Academic medical center	25–34	White/Caucasian	6 to 10
Physician (MD/DO)	Obstetrics and Gynecology	1 to 3	Academic medical center	25–34	Bi-Racial/Multi-Racial, Native American/Alaskan Native, White/Caucasian	6 to 10
Physician (MD/DO)	Obstetrics and Gynecology	4 to 9	Academic medical center	25–34	White/Caucasian	More than 10
Physician (MD/DO)	Obstetrics and Gynecology	4 to 9	Academic medical center	25–34	White/Caucasian	6 to 10
Clinical Nurse Coordinator	Pediatric Infectious Diseases	<1 year	Academic medical center	45–54	White/Caucasian	More than 10
Physician (MD/DO)	Pediatric Infectious Diseases	1 to 3	Academic medical center	35–44	White/Caucasian	More than 10

a BPLWH, birthing people living with HIV who were pregnant or postpartum in the past 12 months.

### Coherence – Do HCWs understand the purpose of the new breastfeeding/chestfeeding guidelines?

Across all HCWs, there was a consensus that the guidelines made sense overall, given the current data, and were in line with best practices in maternal and HIV healthcare. However, opinions differed as to how and when the guidelines should be applied in real-world contexts. For instance, most providers perceived maternal autonomy and informed choice as the primary purpose of the guidelines, with maternal and infant health as a secondary key purpose.

“They empower the mom to, you know, be able to make a choice, which before they didn't have a choice. It was you don't breastfeed. So now they have a choice …My role here is to bring you information about these medical conditions or this decision-making, and then you are the expert in your life and experience” (OB-GYN 6).

“Even for the moms who say, “No, I don't want to breastfeed,” but still being given that choice … Like, hey, this is an option now. Even if you don't want to do it, it's an option” (Pediatric ID Clinical Nurse Coordinator).

Recognition of patient needs and the unique challenges of living with HIV were emphasized in discussions of the importance of maternal autonomy and informed choice for this patient population, in particular, the role of: (1) structural barriers, (2) stigma, (3) breastfeeding/chestfeeding priorities, and (4) the emotional burden of living with HIV.

“I think the guidelines come right out to say that this population faces so much stigma, both external and I think also internal” (Pediatric ID Doctor).

“Patients with HIV experience a lot of other health disparities and discrimination, and advocating for their breastfeeding can also help address some of those other disparities” (OB-GYN 6).

“For a lot of patients who are also ID positive, they also have a lot of social needs going on. And like being HIV positive is one thing they're dealing with, but they're dealing with housing instability. They're dealing their health insurance lapsing. They're dealing with food insecurity” (OB-GYN 7).

The Pediatric ID Doctor emphasized the importance of reducing infant transmission in breastfeeding/chestfeeding more than other interviewees. The Pediatric ID Doctor was the only interviewee to emphasize the burden of an HIV diagnosis and the accompanying medication-related and mental health implications for young people who are vertically infected. Other interviewees mentioned reducing susceptibility to mother-to-child transmission but focused more on maternal autonomy (as noted above).

“This isn't something that everyone should consider. If we can't get viral suppression, if we're not compliant with medications, formula feeding is still probably the best thing to decrease the risk of vertical transmission” (OB-GYN 3).

“Vertically infected young people face a lot of challenges … stigma, both external and internal … because medications have evolved so much, we underestimate, I think, the burden on young people of taking medications every single day … frankly had people die because they cannot, will not take their medication …for some people, the act of taking that pill is a reminder of the fact that they're living with HIV, which is, and that is incredibly upsetting” (Pediatric ID Doctor).

The Disease Intervention Specialist did not emphasize maternal autonomy and informed choice as the guidelines' intention, but rather focused on maternal health and reducing the risk of transmission to the infant. Additionally, the Disease Intervention Specialist acknowledged that there is a choice involved in balancing the risk of transmission with the benefits of breastfeeding/chestfeeding.

“We want them to live the healthiest life that they can. And so, it starts with HIV care … we want to make sure that we're ending the HIV epidemic …we don't want this to be something that's over your head, where I gave my child HIV” (Disease Intervention Specialist).

While the guideline language was considered clear overall, many HCWs wish there were more practical guidance in the guidelines.

“What has been an issue for me with these revised guidelines is that I think that they are so well-intentioned, but they're a little bit high level … At what different levels of maternal detectable viral loads is the risk going to be high enough that people would say we really can't do this? What is the best infant prophylaxis strategy?” (Pediatric ID Doctor).

“I don't want to say they're vague. They're not vague, but they're not really specific. So that sometimes presents a challenge when you when you come up with those …outlier situations” (Pediatric ID Clinical Nurse Coordinator).

Training on the guidelines varied among HCWs: some received formal training, others had no training but were given resources or created their own, and some received training and information from peers or supervision.

“We received training through our fellowship …about HIV and pregnancy …so we have like lectures where we talk about these guidelines” (OB-GYN 1).

“We go to all DIS meetings …And then I just had to do my own personal research” (Disease Intervention Specialist).

“We were provided the resources for kind of our, the like template in terms of how we counsel as our, our, for our division …but there wasn't necessarily a formal training” (OB-GYN 2).

“Watching, you know, my supervisor and the provider, you know, give education on it, then doing it myself” (Pediatric ID Clinical Nurse Coordinator).

### Cognitive participation – are HCWs prepared to engage in and commit to integrating the breastfeeding/chestfeeding guidelines?

Participants reported high levels of commitment to successful guideline implementation, with institutional and collegial backing. They also reported strong motivation to adhere to the guidelines for professional, ethical, and personal reasons. They cited the importance of providing evidence-based care, advocating for patients, and having firsthand experience of what patients themselves experience.

“Our faculty are incredibly supportive and have supported us in learning, advocating, and participating in those discussions” (OB-GYN 6).

“One of the reasons why I went into nursing …my personal experience …I have a real intimate relationship with all of that that I can share and that helps support the moms” (Pediatric ID Clinical Nurse Coordinator).

All participants reported feeling supported in advocating for these guidelines, making them more likely to participate actively in the guidelines. Having champions of the guidelines on one's team and institutional support were cited several times as factors that promoted feelings of empowerment in advocacy.

“I definitely do [feel I can advocate for the guidelines] locally. And I think part of that is the culture of the institution … I think like having faculty come forward as strong supporters of the change within my department, like as somebody who's already inclined, but like the faculty support, so leadership support for the guidelines, I think is really important” (OB-GYN 6).

Participants cited the infrequency of encounters with BPLWH and training limitations as challenges to educating all HCWs who meet BPLWH. Several OB-GYNs cited the challenge of getting all HCWs on the same page regarding supporting birthing parents in breastfeeding/chestfeeding.

“I know from my work on patient experiences with lactation and stuff that I could have a very nuanced discussion in the office, and one comment from a postpartum nurse can undo a lot. So yes, there's plenty of room for cohesion and message with that” (OB-GYN 6).

Participants reported varying strategies and levels of frequency of engaging with the guidelines, including patient-needs-driven guideline references, passive information access such as reading email updates, and actively seeking out updates. Trusted information sources were cited frequently.

“I have the CDC guidelines, and CDC connected bookmarked on my computer. But then also with the, I'm a part of this DIS group, like NCSD [National Coalition of STD Directors]” (Disease Intervention Specialist).

“I'm like subscribed to some various …daily newsletter emails, weekly newsletter emails, but there are like websites that are meant to be like educational and targeted towards like updates in terms of research and guidelines in general” (OB-GYN 2).

“I am on the National Perinatal HIV Guidance website every week. They do update it about once a year … There's a perinatal HIV listserv also that I'm a part of that's run out of UCSF” (Pediatric ID Doctor).

Participants generally found the breastfeeding/chestfeeding guidelines to be straightforward. Several participants employed explanatory strategies and avoided medical jargon.

“I think they're pretty clear …they are concise. They have like a summary at the top, but then they have like, a lot of detail and they're very thorough at the bottom … it's also like pretty clear about what they recommend in bullet point format, which is helpful when you're in clinic” (OB-GYN 1).

“I try to make sure that everything is in like layman's term as possible and try to avoid, like try to define or at least avoid using things that are like medical or jargon. But I think also most of the time, most patients who are HIV positive like are familiar with words like viral load and like undetectable and kind of like discussion about transmission” (OB-GYN 2).

The role of clinical judgment was salient in the interviews, especially regarding patients' ability to follow through with needed testing and the challenges of assessing a suppressed viral load.

“It's easier to recommend breastfeeding if they are more engaged. It gets harder when you've seen them for like three prenatal visits throughout their pregnancy and they've been negative all three visits, but like you haven't really captured them for a long period of time. It's hard to feel secure that like, they're gonna do, they're going to be safe” (OB-GYN 7).

“Our assays detect down to 20. Less than 20 would be undetectable. And I think it ends up being uncomfortable when we have people with kind of borderline viral loads between 20 and 50 …what does suppression really mean in an era of super sensitive assays?” (Pediatric ID Doctor).

### Collective action – Do HCWs feel capable of doing what is needed to fully implement the breastfeeding/chestfeeding guidelines?

Participants who report successful implementation of the guidelines cite co-workers who championed them, resulting in the creation of protocols and working groups. Additionally, Institutional support and team collaboration were strong factors in implementing the guidelines for HCWs.

“Our institution has done this successfully. There are champions in our department and in pediatrics and infectious disease ….I know I just got to refine that path and …done it before …I've never got the sense that we weren't invested in this” (OB-GYN 4).

“Co-workers definitely help. I have a great supervisor …having those DIS meetings …really helps” (Disease Intervention Specialist).

“We've tried to collaborate a lot with adult ID and with MFM over this. And they've been wonderful partners. And I think everyone's really excited about this and really wants to give families a lot of options” (Pediatric ID Doctor).

Participants described cross-specialty differences as stemming from a perceived division of responsibility and differing provider views on who was considered their primary patient. OB-GYNs often initiated counseling during pregnancy but had limited involvement in postpartum management, whereas pediatric clinicians emphasized their responsibility for infant follow-up and the consequences of postnatal transmission. These differing roles appeared to shape both the interpretation and implementation of the guidelines, with OB-GYNs more oriented toward the birthing patient and pediatric clinicians more focused on the infant or family unit. Thus, variation across specialties reflected not only differences in guideline interpretation but also distinct professional responsibilities, clinical priorities, and accountability for outcomes.

“They're not managing these moms and babies after they're born, frankly. So they're having these conversations, but then we're the ones managing. And we're also the ones who are gonna be responsible if a baby does acquire HIV. So I think we take that responsibility really seriously” (Pediatric ID Doctor).

“Maybe from the obstetric side, we're a little maybe more eager to promote breastfeeding, and our pediatric colleagues seem to be a little bit more cautious. And that's just my perception. So, it may not really be true, but that to me signals that like, we're prioritizing different things or interpreting things differently” (OB-GYN 4).

Several OB-GYNs wanted clearer guidance on how to counsel and determine who will ultimately make the decision about breastfeeding/chestfeeding safety, commenting on the challenges of being the first point of contact for counseling while not necessarily being the official decision maker on whether breastfeeding is possible. Institutional challenges to implementation, including the inability to obtain Pediatric ID, Doctors' completion of a pre-birth consult, and differing interpretations of the guidelines, were identified.

“Yeah, I mean, I think that if we aren't all on the same page, it gets really hard to implement anything. Because you can be like, oh, I'm the OB who tells everyone with HIV that they can breastfeed. And that's just not true in practice, right? And so, making sure that everyone is on the page and then having effective handoffs in that phase of care, like after pregnancy is over, making sure there's an effective handoff to the doctors who then take over would be really important” (OB-GYN 3).

“I guess I do wish I had some understanding of what the pediatrician's thought process was, and I don't actually know if somebody's breastfeeding, and we've recommended it because they've been undetectable, and they're going to be able to do it the way that the guidelines say. Does the baby have to be on HAART and things like that. I do I wish I knew some of that so I could pre-counsel my patients since we don't routinely send them to PEDS to get counseling …like is this, I mean, I don't think it's like an automatic like NICU admission or anything like that, but it's there like anything we should be prepping our moms for if we're going to recommend this that like…like the pediatricians could say something different when they like get to postpartum” (OB-GYN 7).

Several participants cited the recency of guideline implementation as a challenge for doctors in fully adopting guideline recommendations. Conflicting interpretations of guidelines and interpersonal resistance from colleagues were frequently mentioned throughout the interviews.

“We have had some colleagues be very, very anxious about this, especially people who for decades were taught that breastfeeding wasn't a safe option for people living with HIV. So we have had situations where prenatally we knew that somebody wanted to breastfeed, their adult HIV doctor supported them, their OB doctors supported them, but then they delivered in an outlying hospital and the community pediatrician had never heard about this before and ended up dissuading the family” (Pediatric ID Doctor).

A few participants cited instances in which providers' decisions did not quite align with guideline practices, and the rationale for those decisions was unclear. Whether due to differing interpretations or priorities, several participants also cited breakdowns in communication and expectation-setting between roles as areas for improvement.

“I don't have a lot of interaction with like the infectious disease team. Who understandably their job is to avoid transmission, right? And the easiest way for that to happen, if the only goal is to avoid transmission, is to formula feed. But that's not the only thing we have to consider. And so I feel like I haven't had that interaction with them to hear their thoughts about it. But those conversations, I think, still need to happen openly between adult infectious disease and pediatrics, honestly” (OB-GYN 3).

Differing interpretations of guidelines lead to differing expectations for BPLWH, which, in turn, place an emotional burden on BPLWH.

“…to have everybody on the same page with their knowledge base. And so that, to reduce mixed messages that patients receive, because that can be really discouraging, or somebody might stop breastfeeding because one person says something. And if that's not evidence-based, it's really hard to come back from, honestly. So, I think broadly, better medical education, especially for what is a physician or APP's role in the overall like support of breastfeeding” (OB-GYN 6).

Peer-to-peer education and reliance on trusted sources of information bolsters relational integration for many participants.

“I get, you know, emails from ACOG about any updates to protocols…one of my partners …is really invested in infectious disease …and so she definitely, anytime there, there are changes to that …she'll update all of us and send out like email updates and those kinds of things, and I obviously try and keep up with the literature as well” (OB-GYN 3).

“I also …just try to kind of basically keep ears out and like listen to what things my colleagues are discussing and my, and attendings and mentors and things like that are discussing to make sure, you know, when people are like, oh, there's, did you hear about this new thing to just try and stay up to date” (OB-GYN 2).

### Reflexive monitoring – how do HCWs judge the effectiveness of guideline implementation in their work?

Participants cited working groups to ensure that institutional-level procedures and processes are discussed across departments and specialties.

“We have working group with PEDS ID. And so will email them and say, this patient is really motivated, she's this many weeks, and we're supposed to notify them, I forget how far along, I'd have to pull it back up. But that's essentially how our practices integrated this is we have this workflow. And so we email that breastfeeding HIV working group, and they determine that the patient is or is not a candidate, usually they are, it's not a problem. And then that comes with the pediatrics consult …If people disagree on their candidacy and that's why we have a working group, and I think that's been addressed nicely” (OB-GYN 5).

Most participants discussed strategies for adapting the guidelines to their roles in the healthcare setting.

“When I've had questions about prophylaxis, one resource is the UCSF perinatal hotline. I've reached out to them to get some input from other providers, and they've also talked a little bit about what other folks are doing. I also have talked to another pediatric infectious disease doctor I have met professionally who's at another institution and their approach is actually pretty different. They're more cautious than we've been with their prophylaxis recommendations” (Pediatric ID Doctor).

“If anybody's ever like, I don't know if I want to breastfeed, I don't push them too hard. Like, it's a hard process. If you're already, if you're worried, like I am happy to give you the resources, but if you don't think that it's gonna be right for you, like I don't feel like it's something that I like necessarily need. It's  …not a hill I'm gonna die on. And it's especially not a hill I'm going to die on for somebody who has additional risk factors and it's going to be a hard battle” (OB-GYN 7).

A recognition of intersectional and systemic vulnerabilities permeated conversations about guideline effectiveness and informed participants' understanding of the unique challenges faced by BPLWH.

“For every family, having a baby makes it harder to do anything, right? Yeah. Going to multiple medical appointments with young children is really, really tough. So I think any appointment for any reason is a big ask” (Pediatric ID Doctor).

“Breastfeeding is really hard and people don't know what to expect. And you know, even when things are going well, women see it as like a big undertaking the first time. And so, you know kind of knowing that patients can have all the normal things that are challenging about breastfeeding plus the stress and the, the work and the time commitment of sort of this extra monitoring, like these are patients who probably just need even more support and preparation” (OBGYN 4).

Patient feedback, primarily through patient emotional responses, was cited by almost all participants as influencing guideline implementation and motivation to adhere to the guidelines.

“I think it's hard for a lot of people, especially when we are so like breast-is-best because they feel like they feel a failure in some way for not being able to do what they're supposed to be doing and what's best for the baby. So, I think she was very upset by that. That she had been told the whole time that, oh, you're doing great. You're taking all your medications. Your viral load is negative. You should definitely breastfeed. It's best for the baby. It's good for bonding between the two of you. And like, that's the whole thing that she heard. And then she was told, no, you shouldn't do it. And I think she felt like kind of like a failure” (OB-GYN 7).

“I think a lot of families are really excited, but a lot of families still have a lot of questions …. I've also disappointed some families who were interested in breastfeeding” (Pediatric ID Doctor).

## Discussion

This qualitative study offers a systematic, cross-disciplinary account from various HCWs of how breastfeeding/chestfeeding guidelines for BPLWH are understood and enacted in clinical practice. Applying NPT revealed that across disciplines, participants perceived the guidelines as conceptually coherent and aligned with contemporary HIV and perinatal care. However, actual implementation was shaped by tensions between maternal autonomy and infant safety, uneven training and exposure, and fragmentation across specialties and care settings.

These findings align with emerging literature indicating that the shift toward shared decision-making in infant feeding for parents with HIV has been broadly welcomed but remains difficult to implement in routine care. Prior work has identified facilitators and barriers to implementation, including institutional support, interdisciplinary collaboration, clinician comfort with counseling, and access to standardized processes, as well as barriers such as residual medicolegal concerns, uncertainty about infant risk, lack of practical guidance, and inconsistent messaging across teams (19–21). Our findings extend this literature by showing how these implementation challenges are experienced across disciplines and how they map onto NPT constructs: clinicians may agree with the recommendations in principle, yet still struggle to enact them consistently when workflows, role boundaries, and local infrastructure are not aligned.

Across interviews, HCWs consistently reported that the guidelines made “logical sense” and were supported by current evidence (NPT construct: coherence). For many OB-GYNs, the guidelines were seen as a tool for reproductive autonomy and equity. Participants explicitly described the new recommendations as addressing historical structural discrimination and longstanding differences between guidance in high-income and low-resource settings (21). At the same time, different professional groups emphasized various aspects of the guidelines; for example, the long-term burdens of pediatric HIV, including complex treatment regimens and stigma, and expressed a heightened sense of responsibility for infant outcomes. These differences suggest that, although the guidelines are broadly understood and accepted, specialties interpret their primary purpose through distinct lenses shaped by whether their core mandate is the adult patient, the infant, or the family unit (20).

Participants generally found the guidelines' language to be clear; however, many described it as too “high-level” for easy application in complex clinical situations, largely due to scientific uncertainty. Questions about operational details, such as specific viral load thresholds, the interpretation of “virologic suppression” in the context of highly sensitive assays, optimal infant prophylaxis strategies, and policies on exclusive vs. mixed feeding, were raised. Without explicit criteria, clinicians relied on clinical judgment and peer consultation. This mirrors published concerns that, while revised guidelines endorse shared decision-making, they leave important operational questions unresolved that clinicians must navigate locally (19, 20, 22). In this context, variability in implementation is unsurprising. Our findings suggest that when national recommendations do not fully specify thresholds or workflows, providers rely on personal experience, local culture, and informal expert consultation, which can amplify inconsistency between specialties and sites. Recent work in pediatric HIV settings similarly suggests that uptake of updated guidance remains uneven and that local interpretation strongly shapes practice (21).

Overall, HCWs demonstrated strong dedication to implementing the guidelines (NPT construct: cognitive participation). Many described a firm, professional, and ethical motivation to provide evidence-based care and to advocate for marginalized patients. Several cited their own clinical experiences, noting that these increased their motivation to help BPLWH access similar opportunities. Institutional culture and visible champions across teams were seen as especially important for justifying the guidelines, supporting advocacy, and encouraging adherence (19, 22). However, preparation and training varied. Some participants reported formal fellowship education or standardized counseling templates. Others relied on self-directed reading, email lists, online resources, and informal peer teaching. Additionally, relatively low patient volumes further limited experiential learning; many providers see BPLWH infrequently, making it difficult to maintain familiarity and confidence. As a result, learning was often patient-driven and episodic, and a small number of challenging cases could disproportionately affect perceptions of risk and feasibility.

Implementation was most successful where organizational structures and interprofessional collaboration were in place (NPT construct: collective action). Participants described local workflows that included identification, counseling, documentation in the electronic health record, and interdisciplinary review involving OB, pediatrics, and ID. These structures helped turn abstract recommendations into consistent practice and reinforced the perception that the institution was committed to supporting breastfeeding/chestfeeding for BPLWH. However, collective action was hindered by fragmentation along the care pathway. OB-GYNs often serve as the first point of counseling, but do not control postpartum decisions about infant prophylaxis, monitoring, and follow-up. Several participants shared cases in which patients, reassured throughout pregnancy that they were good candidates for breastfeeding/chestfeeding based on adherence and viral suppression, were later discouraged from doing so by pediatric colleagues. These inconsistencies could cause more distress and confusion among patients and, for clinicians, potentially undermine trust and the psychological benefits of presenting breastfeeding/chestfeeding as a meaningful choice (20, 21). Importantly, the need for coordination and collaboration across specialties is not unique to infant feeding counseling for BPLWH. Similar challenges are seen in other complex care areas where responsibilities are divided among disciplines with varying clinical priorities, risk thresholds, and accountability structures. The literature on multidisciplinary cancer care, integrated HIV services, and collaborative care models generally indicates that implementation is often hampered when communication is weak, team roles are unclear, and care transitions span organizational or specialty boundaries (23–25). In this broader context, our findings suggest that inconsistent adherence to breastfeeding/chestfeeding guidelines reflects not only HIV-related uncertainties but also a wider systemic challenge of coordinating care across specialties. This perspective helps explain why participants in our study often agreed with the guidelines in theory but faced difficulty implementing them consistently.

Lastly, structural and logistical constraints also limited implementation; for example, geographically-separated services and separate clinic systems can impede joint counseling, which could lead to downstream implications of breastfeeding/chestfeeding (i.e., the duration and intensity of infant antiretroviral therapy, frequency of blood draws, and the impact of insurance and resource instability after pregnancy), which complicates prenatal counseling and expectation-setting. These findings indicate that barriers to implementation are not only informational but also embedded within care pathway design and health systems (19, 21, 26).

HCWs mainly assessed the effectiveness and acceptability of their practice through informal, case-based reflection rather than formal metrics (NPT construct: reflexive monitoring). Providers consulted with working groups, some published work, and colleagues at other institutions when faced with challenging or complex cases and adjusted their practices, as needed. HCWs also acknowledged that breastfeeding/chestfeeding, especially when combined with additional monitoring and appointments for HIV-related care, can impose significant burdens, particularly for patients facing employment constraints, limited resources, childcare demands, and transportation challenges. This illustrates that choices are limited by structural conditions and that even well-intentioned guidelines may unintentionally increase patients' care workload if their implementation does not account for these realities (26).

### Strengths and limitations

A key strength of this study is its use of NPT to systematically examine how new breastfeeding/chestfeeding guidelines for BPLWH are understood and enacted in practice. Applying NPT enabled us to move beyond simple descriptions of attitudes to explore coherence, engagement, collective work, and ongoing appraisal across provider roles. The sample included diverse HCWs involved in perinatal HIV care (OB-GYN, pediatric ID, nursing, and disease intervention specialists), allowing us to capture cross-disciplinary interactions that are often obscured in studies focused on a single specialty. The IDIs provided rich, detailed accounts of how guidelines are interpreted and utilized within real-world institutional contexts.

However, like any study, several limitations should be acknowledged. The research was conducted at a small number of institutions, including academic and specialized settings with existing interest and expertise in perinatal HIV, which may limit its applicability to community or resource-limited settings and could bias the findings toward higher levels of engagement and familiarity with the guidelines. Most HCWs interviewed were OB-GYNs, which is a limitation. The low overall number of BPLWH seen by many participants means that some reflections were based on a few cases or hypothetical scenarios. Data were self-reported and may be affected by recall and social desirability biases, particularly regarding perceived adherence to guidelines and support for patient autonomy. Lastly, we did not include the perspectives of BPLWH themselves in this analysis; future work with this project will incorporate patient experiences to better understand how guideline implementation and mixed messages influence decision-making and well-being.

## Conclusion

HCWs were motivated to support the implementation of the breastfeeding/chestfeeding guidelines and viewed them as an overall positive change. HCWs generally believed the guidelines make sense and use relatively straightforward language. Opinions differed on whether the guidelines need more practical guidance. OB/GYNs were more likely to focus on the benefits for maternal autonomy and informed choice, whereas Infectious Disease HCWs were more likely to focus on the potential impact of vertical infection on the child. Most HCWs cited communication, process, and interpretation challenges between Pediatric Infectious Disease and OB/GYN as barriers to implementation. Taken together with recent work on barriers and facilitators, practice variability, and patient decision-making, our findings suggest that broader normalization of these guidelines will require not only clearer practical guidance but also stronger interdisciplinary coordination, ethical support for counseling, and care models that account for the structural burdens patients face.

## Data Availability

The original contributions presented in the study are included in the article/Supplementary Material, further inquiries can be directed to the corresponding author.
