# Supplementary material for: Evaluating infant feeding practice guidelines for individuals with HIV: a pilot study using qualitative findings with healthcare workers and normalization process theory
Source: Front Reprod Health. 2026 Jul 22;8:1867104. doi: 10.3389/frph.2026.1867104 (PMC13437786; doi:10.3389/frph.2026.1867104)
Supplement: Supplementary file 1 [file Table1.docx]

**Supplementary File: Interview Guide**

**ENGAGE-HIV: Interview Guide for Healthcare Providers**

Thank you for taking the time to participate in this study. I appreciate your willingness to discuss the HIV chest/breastfeeding guidelines. Before we begin, I’d like to take a few moments to explain what to expect and answer any questions you might have.

This study seeks to gain in-depth insights into how U.S. National Guidelines on breast and chest feeding for PLWH are interpreted, applied, and experienced in real-world settings. We are particularly interested in understanding your personal experiences and perceptions—how you interpret the guidelines and what influences your engagement with them. Your insights will be invaluable in identifying the strengths and challenges associated with these practices.

Here’s what to expect during our conversation:

- **Open Dialogue:** We will discuss your thoughts and experiences in a conversational manner. There are no right or wrong answers—your honest perspective is what matters most.
- **Semi-Structured Format:** I have a set of guiding questions, but the conversation will be flexible. If you feel a particular topic is important, please feel free to expand on it.
- **Confidentiality:** Your privacy is our highest priority. Everything you share will be kept confidential, and your responses will be anonymized in any reports or publications. No personal information identifying you will be shared.
- **Voluntary Participation:** Your participation is entirely voluntary, and you are free to skip any question or stop the interview at any time if you feel uncomfortable.
- **Use of Information:** The insights from this interview will help improve how healthcare providers and support systems communicate and implement these guidelines.

After the interview, you will receive a $75 gift card as compensation for your participation. I will need to ask you for some basic information to be able to send the gift card via email. Specifically, your name, email and phone number. We are mandated to ask for a social security number or TIN, but you are not required to provide it. We just have to ask. Let me get that information from you now (INTERVIEWER GATHERS INFO), and you can expect a gift card to arrive at your home within 2 weeks.

Do you have any questions for me? I am going to start recording now. Is that okay with you?

**[BEGIN RECORDING]**

**Section 1: Coherence – Sense-Making of the U.S. National Guidelines for Breast/Chestfeeding for PLHV**

**Understanding the Guidelines**

1. Can you describe, in your own words, what the current HIV chest/breastfeeding guidelines recommend?
   a. **Probe for nurses / doulas / midwives / physicians:** What kind of information would be helpful to your practice around HIV chest/breastfeeding?
   b. **Probe for those less familiar with the guidelines:** How much do you interact with the guidelines?
      i. If you are not familiar with the guidelines, what kind of information would be helpful to have around chest / breastfeeding and HIV?
2. What do you believe are the primary objectives of these guidelines?
   a. **Probe for nurses / doulas / midwives / physicians:** How do these objectives align (or not) with what you see as the most pressing needs of the birthing people you support?
   b. **Probe for those less familiar with the guidelines**: What goals do you think national guidelines on HIV and chest / breastfeeding should focus on?
3. How clear and understandable do you find the language and content of these guidelines?
   a. **Probe for nurses / doulas / midwives / physicians:** When you explain guidelines to patients, what language or examples do you use to make them more accessible?
   b. **Probe for those less familiar with the guidelines:** what types of wording or explanations usually make clinical guidelines easier or harder for you to apply in practice?

**Relevance and Context**

4. How do these guidelines align with your understanding of best practices in HIV care and maternal health?
   a. **Probe for nurses / doulas / midwives / physicians**: Are there traditional or community-informed practices that you draw on, which influence how you interpret these guidelines?
   b. **Probe for those less familiar with the guidelines:** What best practices are you familiar with—if any—around chest / breastfeeding and HIV?
5. In what ways do you think the guidelines address the unique challenges faced by birthing people living with HIV?
   a. **Probe for nurses / doulas / midwives / physicians:** How do the guidelines address the unique challenges that you see as a nurse/doula/midwife for pregnant people living with HIV?
   b. **Probe for those less familiar with the guidelines:** What are some of the unique challenges faced by pregnant people living with HIV? What information would be helpful to address these?
6. Can you identify any areas where the guidelines might be confusing or require further clarification?
   a. **Probe for those less familiar with the guidelines:** For best practices / guidelines you are familiar with, where are areas of confusion around what advice should be given to pregnant people living with HIV around chest / breastfeeding?

**Integration into Practice**

7. How have you integrated the HIV chest/breastfeeding guidelines into your routine practice?
   a. **Probe for nurses / doulas / midwives / physicians**: Can you describe a specific scenario where you had to make a quick decision or adapt care using these guidelines?
   b. **Probe for those less familiar with the guidelines:** What have conversations with pregnant people living with HIV been like around the topic of chest/breastfeeding?
8. What training or resources did you receive that helped you understand these guidelines?
   a. **Probe for nurses / doulas / midwives / physicians**: What additional resources (e.g., visuals, culturally tailored materials, peer learning) would make it easier for you to apply the guidelines?
   b. **Probe for those less familiar with the guidelines:** What kind of training would be helpful to educate you on these guidelines?
9. Do you see any gaps between the guideline recommendations and the realities of your work environment?
   a. **Probe for nurses / doulas / midwives / physicians**: How do you balance guideline recommendations with patients’ personal preferences or any limitations in your setting?
   b. **Probe for those less familiar with the guidelines:** Are there other guidelines you’ve struggled to apply in your setting? What made it difficult?

**Section 2: Cognitive Participation – Engagement and Commitment**

**Commitment to Implementation**

10. What motivates you to adhere to the HIV chest/breastfeeding guidelines in your clinical practice?
    a. **Probe for nurses / doulas / midwives / physicians**: Can you share an example where your commitment to these guidelines directly influenced patient care?
    b. **Probe for nurses / doulas / midwives / physicians**: How do your relationships with patients influence your commitment to following these guidelines?
    c. **Probe for those less familiar with the guidelines:** If you’re not yet using these guidelines, what factors would motivate you to adhere to them?
11. How do you see your role in promoting these guidelines among your colleagues and within your community?
    a. **Probe for nurses / doulas / midwives / physicians**: Do you feel empowered to advocate for these guidelines, and what would make advocacy easier?
    b. **Probe for those less familiar with the guidelines:** If you haven’t promoted them yet, what kind of support or knowledge would help you feel confident doing so?

**Barriers and Facilitators**

12. What institutional factors facilitate your adherence to the guidelines?
    a. **Probe for nurses / doulas / midwives / physicians**: What about interpersonal factors?
    b. **Probe for nurses / doulas / midwives / physicians**: What about personal factors?
    c. **Probe for nurses / doulas / midwives / physicians**: Are there mentorship, supervision, or peer-support systems that make implementation easier?
    d. **Probe for those less familiar with the guidelines**: If you have not or do not consistently implement these guidelines, what factors would you need to do so?
13. Conversely, what challenges or barriers do you face when trying to implement these guidelines?
    a. **Probe for nurses / doulas / midwives / physicians**: Do you encounter resistance from patients, family members, or colleagues? If so, how do you navigate that?
    b. **Probe for those less familiar with the guidelines:** What barriers exist to sharing best practices?
14. How do you overcome obstacles that hinder your ability to fully commit to these guidelines?
    a. **Probe for those less familiar with the guidelines:** How have you worked around barriers when applying other guidelines in the past?

**Section 3: Collective Action – Operationalizing the Work**

**Engagement and Ongoing Participation**

15. How do you stay informed about updates or changes to the guidelines?
    a. **Probe for nurses / doulas / midwives / physicians:** What type of format (e.g., workshops, quick-reference cards, digital platforms) would make staying updated easier in your practice?
    b. **Probe for those less familiar with the guidelines:** Where do you usually get clinical updates?
16. What strategies do you think could enhance engagement among your peers with respect to these guidelines?
    a. **Probe for nurses / doulas / midwives / physicians:** How could professional associations or community-based networks better support engagement?
    b. **Probe for those less familiar with the guidelines:** What types of peer engagement methods usually help you and your colleagues adopt new practices?
17. In your opinion, what role does collaboration (with colleagues, specialists, or community health workers) play in effective guideline implementation?
    a. **Probe for nurses / doulas / midwives / physicians:** How do you see your unique role (e.g., hands-on patient support, advocacy, trust-building) complementing the work of other providers?
    b. **Probe for those less familiar with the guidelines:** How important has collaboration been for you in learning and applying other new practices?

**Section 4: Reflexive Monitoring – Appraisal of the Work**

**Impact on Practice and Patient Outcomes**

18. Have you observed any changes in patient outcomes or behaviors as a result of implementing these guidelines?
    a. **Probe for nurses / doulas / midwives / physicians**: Do you notice differences in how patients respond depending on who delivers the information (nurse, doula, midwife, physician)?
    b. **Probe for those less familiar with the guidelines:** What kinds of changes in patient outcomes would you want to see in this area?
19. How do you evaluate the success of the guidelines in your practice?
    a. **Probe for nurses / doulas / midwives / physicians**: What patient or family feedback has been most meaningful in shaping how you view the effectiveness of the guidelines?
    b. **Probe for those less familiar with the guidelines:** How do you usually measure whether a new guideline or practice is working well with your patients?
20. What feedback do you receive from patients regarding these practices, and how does it affect your commitment to the guidelines?
    a. **Probe for nurses / doulas / midwives / physicians**: How do patients’ emotional responses (e.g., trust, fear, relief) influence your approach to guideline implementation?
    b. **Probe for those less familiar with the guidelines:** What types of patient reactions to care have influenced your commitment in other areas?

**[STOP RECORDING]**

Do you know anyone who you’d recommend to participate in this study? That could be healthcare workers engaging with birthing parents living with HIV or Birthing Parents Living with HIV themselves. If so, please connect them with us!
