# Supplementary material for: Evaluating infant feeding practice guidelines for individuals with HIV: a pilot study using qualitative findings with healthcare workers and normalization process theory
Source: Front Reprod Health. 2026 Jul 22;8:1867104. doi: 10.3389/frph.2026.1867104 (PMC13437786; doi:10.3389/frph.2026.1867104)
Supplement: Supplementary file 2 [file Table2.docx]

# **Supplementary File 2. Additional Quotations from Participants**

| **Theme** | **Illustrative Quotes** |
| --- | --- |
| Coherence – Do HCWs understand the purpose of the new breastfeeding/chestfeeding guidelines? | *"A lot times when people are HIV positive, it's not their only problem on their problem list. And so the 15-minute appointment, and you're like, okay, are you taking your... Oh wait, you also have chronic hypertension and you're a diabetic...how's, how was your anticoagulation? Cause you had a stroke last year. Like there are so many other things to talk about...that I think that that's something that per take, like doesn't like, you know, asking those things where like, okay, we'll refer you to social work...and just like hope that like everything gets tied up in a bow" (OB-GYN 7).* |
|  | *“The primary objective is to help providers and patients reduce mother-to-child transmission of HIV while also promoting other health goals such as breastfeeding, bonding, food security, things like that” (OB-GYN 1).* |
|  | *“I think the part that's just confusing is like how, how to monitor, like once patients are choosing to undergo that and the baby's here, like what kind of monitoring should be recommended. And...our pediatric infectious disease team...I don't know if these guidelines they use are from, you know, their governing body or anything like that, but it certainly isn't outlined in our, you know, our guidelines that I've seen” (OB-GYN 3).* |
| Cognitive Participation – are HCWs prepared to engage in and commit to integrating the breastfeeding/chestfeeding guidelines? | *“I think that we have an obligation to, to follow guidelines and provide evidence-based care” (OB-GYN 1).* |
|  | *“Part of the challenge in implementing these guidelines sounds like it's not having [BPLWH] frequently enough” (OB-GYN 4).* |
|  | *“I have no formal process...if someone presents something, that's great...But a lot [of] it is like, gosh, I've got a patient tomorrow. Let me just look up and make sure nothing has, has changed. So it's driven by the patient that I'm seeing” (OB-GYN 4).* |
|  | *“I whenever I have a patient, I like read, I go through [the guidelines] again... I use...HIV.gov...I look at that website, you know, periodically to see if there's been any changes to other aspects of HIV care” (OB-GYN 1).* |
|  | *“I think that breastfeeding is something really important. And so I just care about it from that principle. I also like to find that one of the coolest things about HIV care is how drastically it's improved and how amazing the outcomes are for patients now” (OB-GYN 4).* |
|  | *“All it takes is one side comment too about, ‘oh, you’re breastfeeding?’ and then now she, you know, and you've lost a lot of progress...In the postpartum floor...sometimes people are not as encouraging or questioning" (OB-GYN 5).* |
|  | *“…if they're, you know, desiring to chest feed, then, and they're motivated to do it, then they will likely stay compliant with their medications, but, you know, I choose to believe in the best in my patients” (OB-GYN 3).* |
| Collective Action – Do HCWs feel capable of doing what is needed to fully implement the breastfeeding/chestfeeding guidelines? | *“The division department I work in is pretty good about kind of trying to keep consistent practice across our division” (OB-GYN 2).* |
|  | *“From a pediatric perspective, we...Really don't want to have any instances of an infant acquiring HIV. And of course, nobody wants that. But I think it's a little bit different as an adult provider, used to doing more adult counseling and joint decision-making with adult patients and really seeing themselves as somebody who needs to give their patients all the options, versus in pediatrics, where our immediate focus is always the child” (Pediatric ID Doctor).* |
|  | *“It's one thing for me to say, ‘I think you're a good candidate,’ but they are the ones that, you know, actually have to do all this follow-through, and the Pediatrician has to make sure that the baby is not at risk for being exposed, you know” (OB-GYN 4).* |
|  | *“I don't know well enough what's gonna be asked of the birthing parent, the chestfeeding parent during the breastfeeding relationship. And, you know, does that change how long babies are gonna have to be on medication? Is it gonna, how long, how often are we gonna have blood draws and some of these patients lose their insurance once they're no longer pregnant? And how are they gonna afford their medications to continue longer or their lab draws, and those kinds of things, that I think is the hardest, the biggest barrier” (OB-GYN 3).* |
|  | *"That's the issue. It's like, they [peds] usually don't see them until the baby's born. Oftentimes, they don't see PEDS beforehand. Like we usually like, PED will see moms if there is like a fetal anomaly or something like that to discuss what that looks like after,...but PEDS usually do not counsel moms antepartum... I know it's probably worth having conversations beforehand. I think it's just like our, our clinics aren't co-located and like, it's not just something that we routinely refer people to” (OB-GYN 7).* |
|  | *“I know that we have one person in our like MFM department who's really passionate about…about ID positive moms...she's given us, like, maybe a couple of talks over the past…four years...I don't know that I've ever actually pulled up and read the guidelines myself. I think most of the teaching has probably come through her and her interpretation of the guidelines for us... one of our high-risk doctors who's like particularly passionate and has given us a couple of lectures. I don't know that like I could reliably find her slides...but those are theoretically accessible to us...on like our, our SharePoint for our residency” (OB-GYN 7).* |
|  | *“Pediatricians, they are focused maybe a bit more on ensuring there's no transmission, and you know, I've, we've had some discrepancies, and I, this is beyond the guidelines themselves, but more expert opinion of...You know, why do we need to exclusively breastfeed? You know? You can't have a combination. What's the underlying reason for that?... I don't know that there's a lot of consensus on that” (OB-GYN 5).* |
|  | *“I'm not taking care of baby postpartum. Like what are my concerns and what should my counseling points be?” (OB-GYN 7).* |
|  | *“I still think you have a cohort of providers coming up who have traditionally been taught HIV is a hard stop for breastfeeding. And so I don't know that, and that will change probably in five years, but they are strong members of the team that, I mean, shoot, what if clinics are busy and I and the attending forget? Because this is also new. And so I think it's, you know, just continuing to educate the whole team that this is the new guidelines, recommendations, and something that we're offering” (OB-GYN 5).* |
|  | *“I know we've had a couple of patients who like have a strong desire to breastfeed and have been like compliant on, you know, their Biktarvy or whatever they're taking throughout their pregnancy. And then it seems like, like I know that I had like one specific patient who was like suppressed through her entire pregnancy the last time, and really wanted to breastfeed, and I was seeing her for her subsequent pregnancy. And she was like, yeah, and then I got up to the postpartum floor and the pediatrician told me that, like... I shouldn't do it. And I don't know what that conversation was like. Like if she was like, oh, well, I work, you know, six days a week for 14 hours. So, I'm planning to breastfeed at some points and formula feed at some point. And they were like, okay, well we recommend only exclusive breastfeeding. So, we would recommend you not. Like, I don't know if that was the conversation that happened, but for somebody who like had a desire to breast feed and was like viral load negative and throughout pregnancy and that's like. You know, as her OBs, what we had been telling her the whole pregnancy that she needed to have to breastfeed, then I think it was like confusing for her that like, okay, then the PEDS team just like shut me down” (OB-GYN 7).* |
|  | *“I don't think it's fair to them to, you know, deliver and…then you're a day postpartum and you have this expectation in your head that like, oh, I'm going to breastfeed and you know if PEDS comes in there and they're like, Hey, I'm gonna go draw the blood or we're all we're going to give you... I forget what antiretroviral they use, but Zidovudine or whatever it is, I haven't prepared that family for what that might look like, or I haven't asked PEDS to come in and have a consult to discuss… I mean, it's the root of most conflict, I feel like it's a different set of expectations, and then patients have a tough time” (OB-GYN 5).* |
|  | *“Ones that I'm tracking, I'll check back on, you know, somewhat frequently...you know, we're lucky with SMFM and ACOG...And then I guess maybe one of the best ways is you just talk to people about work, I guess, for better and for worse. And so I learned a lot from, you know, my coworkers and just, hey, did you see that new thing?” (OB-GYN 5)* |
| Reflexive Monitoring – How do HCWs judge the effectiveness of guideline implementation in their work? | *“I don't necessarily feel like we do a good job taking into consideration those factors when we counsel people on breastfeeding. I think being at a baby-friendly hospital, the line is, you should try to breastfeed. We have lactation support available to help you. And I think for a lot of people who have never breastfed a baby, we're like, oh yeah, as long as you get in the latch, you'll be fine. That's the hardest part. And then I don't routinely... having been somebody who has breastfed a baby, I know how intense it can be, but I also don't routinely ask my patients, is your job going to support you in taking a break every three hours for 15 minutes? When are you going back to work? I do ask them if they have a breast pump. Do you have support at home? Do you, have a deep freezer? Like, do you, like, do you have all that you're gonna need?” (OB-GYN 7).* |
|  | *“The PEDS doctor you work with, having a fear or maybe their fear or their anxiety around it, can influence the decision-making process of the mom. For better or for worse” (Pediatric ID Clinical Nurse Coordinator).* |
|  | *“When we're talking about, you know, those kind of questionable areas, where the viral load is kind of, maybe close to that cut off point, or like, you know, it's like, Oh, okay, well I've you know, I gotta talk to somebody, you know, like a doctor about this. Or the nutritionist” (Pediatric ID Clinical Nurse Coordinator).* |
